# Sex Differences in Heart Failure With Preserved Ejection Fraction

**DOI:** 10.1161/JAHA.120.018574

**Published:** 2021-02-23

**Authors:** Yohei Sotomi, Shungo Hikoso, Daisaku Nakatani, Hiroya Mizuno, Katsuki Okada, Tomoharu Dohi, Tetsuhisa Kitamura, Akihiro Sunaga, Hirota Kida, Bolrathanak Oeun, Taiki Sato, Sho Komukai, Shunsuke Tamaki, Masamichi Yano, Takaharu Hayashi, Akito Nakagawa, Yusuke Nakagawa, Yoshio Yasumura, Takahisa Yamada, Yasushi Sakata, the PURSUIT‐HFpEF Investigators

**Affiliations:** ^1^ Department of Cardiovascular Medicine Osaka University Graduate School of Medicine Osaka Japan; ^2^ Department of Social and Environmental Medicine Osaka University Graduate School of Medicine Osaka Japan; ^3^ Division of Biomedical Statistics Department of Integrated Medicine Graduate School of Medicine Osaka University Osaka Japan; ^4^ Division of Cardiology Osaka General Medical Center Osaka Japan; ^5^ Division of Cardiology Osaka Rosai Hospital Osaka Japan; ^6^ Cardiovascular Division Osaka Police Hospital Osaka Japan; ^7^ Division of Cardiology Amagasaki Chuo Hospital Hyogo Japan; ^8^ Department of Medical Informatics Osaka University Graduate School of Medicine Suita Japan; ^9^ Division of Cardiology Kawanishi City Hospital Hyogo Japan

**Keywords:** diastolic dysfunction, heart failure, preserved left ventricular function, prognosis, sex, Heart Failure

## Abstract

**Background:**

The female preponderance in heart failure with preserved ejection fraction (HFpEF) is a distinguishing feature of this disorder, but the association of sex with degree of diastolic dysfunction and clinical outcomes among individuals with HFpEF remains unclear.

**Methods and Results:**

We conducted a prospective, multicenter, observational study of patients with HFpEF (PURSUIT‐HFpEF [Prospective Multicenter Observational Study of Patients with Heart Failure with Preserved Ejection Fraction]: UMIN000021831). Between 2016 and 2019, 871 patients were enrolled from 26 hospitals (follow‐up: 399±349 days). We investigated sex‐related differences in diastolic dysfunction and postdischarge clinical outcomes in patients with HFpEF. The echocardiographic end point was diastolic dysfunction according to American Society of Echocardiography/European Association of Cardiovascular Imaging criteria. The clinical end point was a composite of all‐cause death and heart failure readmission. Women accounted for 55.2% (481 patients) of the overall cohort. Compared with men, women were older and had lower prevalence rates of hypertension, coronary artery disease, and chronic kidney disease. Women had diastolic dysfunction more frequently than men (52.8% versus 32.0%, *P*<0.001). The incidence of the clinical end point did not differ between women and men (women 36.1/100 person‐years versus men 30.5/100 person‐years, *P*=0.336). Female sex was independently associated with the echocardiographic end point (adjusted odds ratio, 2.839; 95% CI, 1.884–4.278; *P*<0.001) and the clinical end point (adjusted hazard ratio, 1.538; 95% CI, 1.143–2.070; *P*=0.004).

**Conclusions:**

Female sex was independently associated with the presence of diastolic dysfunction and worse clinical outcomes in a cohort of elderly patients with HFpEF. Our results suggest that a sex‐specific approach is key to investigating the pathophysiology of HFpEF.

**Registration:**

URL: https://upload.umin.ac.jp; Unique identifier: UMIN000021831.

**Nonstandard Abbreviations and Acronyms**
HFpEF: heart failure with preserved ejection fraction

**Clinical Perspective**

**What Is New?**

- In patients with acute heart failure and preserved left ventricular ejection fraction from the PURSUIT‐HFpEF (Prospective Multicenter Observational Study of Patients with Heart Failure with Preserved Ejection Fraction) prospective multicenter East‐Asian real‐world heart failure with preserved ejection fraction registry, female sex was independently associated with the presence of echocardiographic diastolic dysfunction according to American Society of Echocardiography/European Association of Cardiovascular Imaging criteria.
- Although the incidence of the clinical end point did not differ between women and men, female sex was independently associated with increased risk of the clinical end point after multivariable adjustment.

**What Are the Clinical Implications?**

- Sex differences in heart failure with preserved ejection fraction suggest the need for further research to better understand underlying pathophysiology, including contributions of sex hormones and sex hormone deficiency, and thereby identify novel preventive and disease‐modifying treatments for heart failure with preserved ejection fraction.

Epidemiological studies have established that patients with heart failure with preserved ejection fraction (HFpEF) are more likely to be female than male. Women accounted for only 20% to 25% of subjects in clinical trials evaluating heart failure with reduced ejection fraction,^1^, ^2^, ^3^ whereas in clinical trials assessing HFpEF, women account for as many as 50% to 60% of the trial cohort.^4^, ^5^ Female sex predominance is one of the strongest distinguishing features of HFpEF compared with heart failure with reduced ejection fraction or other cardiovascular disease.

The immune system and inflammation have been thought to be central to the development of HFpEF.^6^ Several comorbidities, including hypertension, diabetes mellitus, atrial fibrillation, obesity, and ischemia, are known to be associated with development and prognosis of HFpEF. Inflammation driven by such comorbidities may be a fundamental mechanism causing myocardial dysfunction. Impacts of the comorbidities differ between women and men. For instance, hypertension increases the risk of heart failure (HF) by 3× in women, compared with 2× in men.^7^ Diabetes mellitus has a more pronounced effect on HF in women, increasing the HF risk 5× in women compared with 2.4× in men.^8^ Atrial fibrillation increases the risk of HF hospitalization 1.63× in women as compared with 1.37× in men.^9^ Women have stronger immune responses than men, which may contribute to the different impacts on the development of diastolic dysfunction and subsequent clinical outcomes between the sexes.^10^

Exploring mechanisms behind the sex differences in HFpEF may help us to understand underlying HFpEF pathophysiology and to identify more specific therapeutic approaches. The purpose of the present study was to assess sex differences in the prevalence of diastolic dysfunction and clinical outcomes in HFpEF.

## Methods

Our study data will not be made available to other researchers for purposes of reproducing the results because of institutional review board restrictions.

### Study Patients

The PURSUIT‐HFpEF (Prospective Multicenter Observational Study of Patients with Heart Failure with Preserved Ejection Fraction) study is a prospective, multicenter, observational study in which collaborating hospitals in Osaka record clinical, echocardiographic, and outcome data of patients with acute decompensated heart failure with preserved left ventricular ejection fraction (≥50%) (UMIN‐CTR [University Hospital Medical Information Network Clinical Trials Registry] ID: UMIN000021831). Consecutive patients with acute decompensated heart failure and preserved ejection fraction were prospectively registered and agreed to be followed up for collection of outcome data. Acute decompensated heart failure was diagnosed on the basis of the following criteria: (1) clinical symptoms and signs according to the Framingham Heart Study criteria^11^; and (2) serum NT‐proBNP (N‐terminal pro‐B‐type natriuretic peptide) level of ≥400 pg/mL or BNP (brain natriuretic peptide) level of ≥100 pg/mL. All patients provided written informed consent for participation in this study. The study protocol was approved by the ethics committee of each participating hospital. This study conformed to the ethical guidelines outlined in the Declaration of Helsinki.

Details of the data collection have been described elsewhere.^12^, ^13^ In brief, basic patient characteristics, echocardiography, laboratory tests, and lists of medications were obtained on admission, at discharge, and at each annual follow‐up time point. We used laboratory data and echocardiography data at the time of discharge (in stable condition after treatment of acute decompensated heart failure) in this analysis.

### Study Design and End Points

The present study aimed to assess the frequency of diastolic dysfunction in women and men and to investigate the sex‐related differences in causes of diastolic dysfunction and prognostic predictors for postdischarge clinical outcomes in patients with HFpEF. Sex, systemic inflammation represented by C‐reactive protein, and various basic comorbidities were comprehensively evaluated in order to estimate their impacts on diastolic dysfunction and postdischarge clinical outcome.

The echocardiographic end point was diastolic dysfunction.^14^ Based on the echocardiographic data obtained at discharge, diastolic dysfunction was diagnosed according to the American Society of Echocardiography and European Association of Cardiovascular Imaging (ASE/EACVI) guidelines for diastolic function assessment.^14^ The 4 recommended variables for identifying diastolic dysfunction and their abnormal cutoff values are: septal e' <7 cm/s or lateral e' <10 cm/s, average E/e' ratio >14, left atrial volume index >34 mL/m^2^, and peak tricuspid valve regurgitation velocity >2.8 m/s. Only patients with all 4 criteria available were analyzed. Left ventricular diastolic dysfunction was diagnosed if >50% of the parameters met these cutoff values.

The clinical end point was a composite of all‐cause death and heart failure readmission. All patients were followed up in each hospital after discharge. Survival data were obtained by dedicated coordinators and investigators by direct contact with patients and their physicians at the hospital or in an outpatient setting or by a telephone interview with their families or by mail. In the present analysis, we analyzed all available clinical follow‐up data up to the end of 2019.

### Statistical Analysis

Data are presented with listwise deletion. Categorical variables are expressed as counts (percentages) and compared with the chi‐square test or Fisher's exact test. Continuous variables are expressed as mean (SD) or median (interquartile range) and compared using Student *t* test or the Mann–Whitney *U* test as appropriate. The clinical end point (a composite of all‐cause death and heart failure readmission) was assessed according to sex in a time‐to‐first‐event fashion with the Kaplan–Meier method and compared with the log‐rank test. Impact of female sex on the echocardiographic and clinical end points was assessed with a binary logistic regression model and the Cox proportional hazards model, respectively. Sex was the variable of interest and the other covariates in the models were as follows: C‐reactive protein, age, anemia (hemoglobin level <12 g/dL in women and <13 g/dL in men according to the World Health Organization definition^15^), hypertension, diabetes mellitus, dyslipidemia, coronary artery disease, chronic kidney disease, atrial fibrillation, obesity (body mass index ≥25), and cholinesterase.^6^, ^13^ These covariates were chosen based on the clinical consensus and our previous reports.^6^, ^13^ Because we aimed to investigate the fundamental sex‐related pathophysiology, we included only basic characteristics in the covariates. However, as a sensitivity analysis, we additionally constructed a Cox proportional hazards model for the clinical end point that included the aforementioned comorbidities and postdischarge medications with prescription rates that were significantly different between women and men. The presence of a statistically significant interaction between sex and the model covariates was tested by the Wald test. An interaction term between each covariate and sex was included in the multivariable models to identify sex‐related differences in predictors of the echocardiographic and clinical end points. Adjusted probability curves in women and men were created with this model. The proportional hazards assumption of sex for the clinical end point was confirmed by Schoenfeld residuals (*P*=0.67). The influence of these factors on the echocardiographic and clinical end points were also assessed in women and men separately in order to investigate the sex differences in causes and prognostic factors of HFpEF. As additional analyses, we evaluated the association between the aforementioned covariates and individual components of the clinical end point. The Cox proportional hazards model was used for all‐cause death. The Fine and Gray model was used for heart failure readmission considering all‐cause death as a competing risk.^16^ A *P*<0.05 was considered statistically significant. The significance level for subgroup analysis (women and men) was 0.025 after adjustment for multiplicity using the Bonferroni correction. All analyses were undertaken using SPSS 24.0 (IBM Corporation, Armonk, NY) or R software (version 3.6.2; R Foundation for Statistical Computing, Vienna, Austria).

## Results

### Study Subjects

Between June 2016 and December 2019, 871 patients were enrolled from 26 hospitals. Mean follow‐up duration was 399±349 days. Patients' characteristics are tabulated in Table 1.^17^ Of 871 patients enrolled, 481 (55.2%) were women and 389 (44.7%) were men. A single patient with missing sex data was excluded from the entire analysis. Compared with men, women were older; had lower prevalence rates of hypertension, coronary artery disease, and chronic kidney disease; and were less commonly smokers. The level of C‐reactive protein was lower in women than in men. There was no significant difference in body mass index, NT‐proBNP, or prevalence of dyslipidemia, diabetes mellitus, or atrial fibrillation. During hospitalization, a diagnosis of cardiac amyloidosis was made in 5 patients (women 0/481 [0%] versus men 5/389 [1.3%], *P*=0.013). Medications at discharge are presented in Table 2. Angiotensin II receptor blockers, calcium channel blockers, and antiplatelet drugs were more frequently used in men than in women.

**Table 1:** Patient Characteristics

Variable	Women	Men	*P* Value
Number	481*	389*	
Age, y	82.23 (8.63)	79.75 (8.93)	<0.001
Body mass index	21.73 (4.82)	22.19 (3.80)	0.124
Body weight, kg	47.77 (11.81)	58.92 (11.49)	<0.001
Obesity (body mass index ≥25)	90 (19.0)	82 (21.4)	0.387
Systolic blood pressure, mm Hg	118.61 (18.12)	119.44 (17.88)	0.502
Diastolic blood pressure, mm Hg	66.20 (11.91)	65.34 (12.01)	0.296
Heart rate, bpm	72.56 (13.47)	70.09 (13.13)	0.007
NYHA class			0.011
NYHA I	153 (32.4)	155 (40.4)	
NYHA II	268 (56.8)	207 (53.9)	
NYHA III	43 (9.1)	17 (4.4)	
NYHA IV	8 (1.7)	5 (1.3)	
Frail^†^	186 (38.8)	73 (18.8)	<0.001
HFA‐PEFF score			0.758
Low (0–1)	6 (1.3)	4 (1.1)	
Intermediate (2–4)	132 (29.0)	115 (31.3)	
High (5–6)	317 (69.7)	248 (67.6)	
History			
Hypertension	395 (82.5)	340 (87.6)	0.037
Dyslipidemia	207 (43.3)	149 (38.7)	0.186
Diabetes mellitus	149 (31.2)	138 (35.9)	0.147
Anemia	330 (68.8)	284 (73.4)	0.136
Atrial fibrillation	178 (37.2)	153 (39.3)	0.528
Persistent atrial fibrillation	145 (81.9)	128 (85.9)	0.368
Paroxysmal atrial fibrillation	32 (18.1)	21 (14.1)	
Congestive heart failure, hypertension, age ≥ 75 years, diabetes mellitus, stroke or transient ischemic attack, vascular disease, age 65–74 years, sex category score	5.27 (1.16)	4.46 (1.23)	<0.001
Congestive heart failure, hypertension, age > 75 years, diabetes mellitus, previous stroke score	3.24 (1.06)	3.32 (1.04)	0.265
Smoking			<0.001
Nonsmoker	392 (82.7)	144 (37.8)	
Current smoker	26 (5.5)	61 (16.0)	
Past smoker	56 (11.8)	176 (46.2)	
Bleeding	17 (3.6)	22 (5.8)	0.139
Prior hospitalization for heart failure	116 (24.7)	97 (25.6)	0.811
Hypertrophic cardiomyopathy	20 (4.3)	12 (3.2)	0.471
Secondary cardiomyopathy	8 (1.7)	5 (1.3)	0.782
Family history of heart failure	27 (6.2)	9 (2.6)	0.016
Atrioventricular block	33 (7.0)	35 (9.2)	0.254
Sick sinus syndrome	41 (8.8)	23 (6.1)	0.152
Pacemaker implantation	42 (8.8)	26 (6.7)	0.310
Pericardial disease	5 (1.1)	5 (1.3)	0.760
Coronary artery disease	57 (12.1)	93 (24.2)	<0.001
Percutaneous coronary intervention	44 (9.2)	75 (19.4)	<0.001
Coronary artery bypass graft	10 (2.1)	21 (5.4)	0.010
Myocardial infarction	17 (3.6)	48 (12.6)	<0.001
Open heart surgery	35 (7.3)	29 (7.5)	>0.999
Peripheral artery disease	18 (3.9)	30 (8.0)	0.011
Chronic kidney disease	163 (34.2)	177 (46.0)	<0.001
Dialysis	2 (0.4)	12 (3.1)	0.002
Stroke	62 (13.1)	59 (15.3)	0.375
Liver dysfunction	27 (5.6)	29 (7.6)	0.269
Malignant tumor	39 (8.2)	60 (15.7)	0.001
Laboratory data			
Hemoglobin, g/dL	11.2 (1.96)	11.7 (2.09)	<0.001
Hemoglogin A1c, %	6.15 (0.90)	6.14 (0.89)	0.979
Creatinine, mg/dL	1.00 [0.80, 1.40]	1.20 [1.00, 1.70]	<0.001
Estimated glomerular filtration rate, mL/min per 1.73 m^2^	41.89 (18.66)	43.89 (21.52)	0.146
High‐density lipoprotein, mg/dL	45.36 (11.69)	42.53 (13.16)	0.002
Low‐density lipoprotein, mg/dL	96.92 (28.95)	92.09 (31.34)	0.029
Total cholesterol, mg/dL	165.43 (35.37)	155.94 (35.03)	<0.001
Triglyceride, mg/dL	108.00 (45.91)	103.45 (48.57)	0.186
Cholinesterase, IU/L	221.84 (69.55)	204.58 (65.85)	0.001
C‐reactive protein, mg/dL	0.27 [0.11, 0.75]	0.31 [0.13, 1.16]	0.036
NT‐proBNP, pg/mL	1090 [481, 2340]	1090 [489, 2590]	0.955

Data with listwise deletion are expressed as mean (SD), median [interquartile range], or number (percentage).

* A single patient with missing sex data was excluded from the entire analysis. HFA‐PEFF indicates Heart Failure Association‐pretest assessment, echocardiography and natriuretic peptide, functional testing, final etiology; NT‐proBNP, N‐terminal pro‐B‐type natriuretic peptide; NYHA, New York Heart Association.

^†^ Frail was defined as the clinical frailty scale score ≥5.^17^ HFA‐PEFF score is a diagnostic scoring system for heart failure with preserved ejection fraction recommended by the Heart Failure Association of the European Society of Cardiology.^18^

**Table 2:** Medication at Discharge

Variable	Women	Men	*P* Value
Number	481*	389*	
Angiotensin‐converting enzyme inhibitors	78 (16.4)	80 (20.8)	0.111
Angiotensin II receptor blockers	158 (33.1)	158 (40.9)	0.019
Beta blockers	262 (54.9)	210 (54.5)	0.945
Calcium channel blockers	207 (43.4)	206 (53.5)	0.003
Mineralocorticoid receptor antagonists	182 (38.2)	144 (37.3)	0.832
Diuretics	380 (79.7)	317 (82.1)	0.386
Vasodilators	33 (6.9)	45 (11.7)	0.017
Digitalis	19 (4.0)	11 (2.9)	0.456
Oral hypoglycemic agents	28 (5.9)	38 (9.9)	0.039
Sodium‐glucose transport protein 2 inhibitors	23 (4.8)	19 (4.9)	>0.999
Statins	165 (34.6)	124 (32.2)	0.469
Anti‐arrhythmic drugs	48 (10.1)	23 (6.0)	0.034
Anticoagulants	276 (57.9)	229 (59.3)	0.677
Antiplatelet drugs	118 (24.7)	143 (37.1)	<0.001

Data with listwise deletion are expressed as number (percentage).

* A single patient with missing sex data was excluded from the entire analysis.

### Echocardiographic End Point

**Figure 1:**
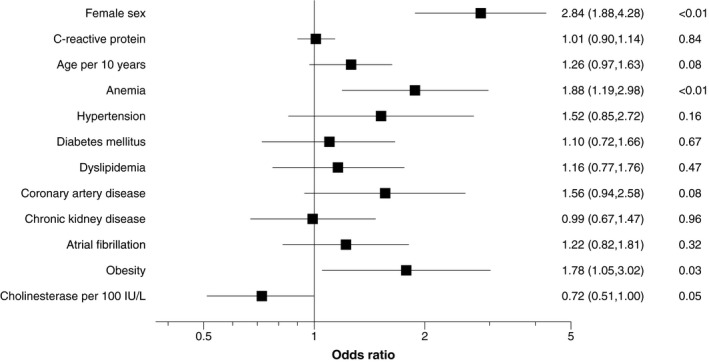
Comorbidities related to diastolic dysfunction in the overall cohort. Multivariable binary logistic regression analysis was performed in order to assess the impact of multiple comorbidities on the echocardiographic end point (diastolic dysfunction) in the overall cohort (N=595). Results are illustrated as a forest plot. Female sex, anemia, and obesity were significant factors associated with diastolic dysfunction. OR indicates odds ratio.

The echocardiographic data in the present cohort were overall in the normal range, except for left atrial parameters and left ventricular mass (Table 3).^19^ The left atrial parameters and left ventricular mass were substantially larger than the normal values of the Japanese cohort.^19^ A total of 595 patients had enough echocardiographic data for the assessment of diastolic dysfunction at discharge. Of these patients, 261 (43.9%) had diastolic dysfunction according to the ASE/EACVI criteria at discharge. Its prevalence was significantly higher in women than in men (179 [52.8%] versus 82 [32.0%], *P*<0.001). In the overall cohort, female sex, anemia, and obesity were independent factors associated with diastolic dysfunction (Figure 1). In women, anemia was a unique and significant associated factor, whereas in men, there was no significant independent factor associated with diastolic dysfunction. However, the sex subgroup analysis did not show significant interactions between the effect of the individual factors and sex (Figure S1).

**Table 3:** Echocardiographic Data

Variable	Women	Men	*P* Value
Number	481*	389*	
Left atrial diameter, mm	43.98 (8.67)	44.34 (8.03)	0.546
Left atrial volume index^†^	58.03 (32.76)	51.02 (24.56)	0.002
Left ventricular diastolic diameter, mm	43.87 (5.87)	47.84 (6.35)	<0.001
Left ventricular systolic diameter, mm	28.19 (4.83)	31.55 (5.89)	<0.001
Left ventricular ejection fraction^†^, %	61.21 (7.79)	59.60 (7.76)	0.006
left ventricular fractional shortening, %	35.81 (6.03)	34.22 (6.78)	<0.001
Left ventricular outflow tract diameter, mm	19.10 (1.97)	21.09 (1.91)	<0.001
Interventricular septum thickness, mm	9.72 (2.16)	10.35 (2.12)	<0.001
Left ventricle posterior wall thickness, mm	9.66 (2.13)	10.33 (1.93)	<0.001
Left ventricular mass, g	145.5 (56.2)	180.4 (55.6)	<0.001
Left ventricular mass index, g/m^2^	104.4 (35.5)	111.3 (33.9)	0.006
Relative wall thickness	0.45 (0.11)	0.44 (0.11)	0.395
Peak A velocity, m/s	0.85 (0.28)	0.81 (0.25)	0.079
Peak E velocity, m/s	0.87 (0.34)	0.82 (0.30)	0.012
Deceleration time, s	0.22 (0.07)	0.21 (0.07)	0.692
E/A ratio	1.02 (0.65)	1.00 (0.60)	0.794
lateral a', m/s	0.08 (0.03)	0.09 (0.03)	0.062
septal a', m/s	0.07 (0.02)	0.08 (0.02)	0.006
lateral e', m/s	0.07 (0.03)	0.08 (0.03)	0.001
septal e', m/s	0.05 (0.02)	0.06 (0.02)	<0.001
E/e' (mean)	15.24 (7.01)	12.71 (5.87)	<0.001
Right ventricle diastolic diameter	31.35 (6.42)	33.71 (6.91)	<0.001
Tricuspid annular plane systolic excursion, mm	17.09 (4.41)	18.04 (4.63)	0.005
Tricuspid valve regurgitation pressure gradient, mm Hg	29.17 (10.27)	26.95 (8.22)	0.002
Aortic valve regurgitation			
None	176 (38.3)	149 (40.5)	0.916
Trace	115 (25.1)	84 (22.8)	
Mild	135 (29.4)	108 (29.3)	
Moderate	32 (7.0)	27 (7.3)	
Severe	1 (0.2)	0 (0.0)	
Aortic valve stenosis			
None	405 (88.2)	334 (90.8)	0.149
Mild	33 (7.2)	25 (6.8)	
Moderate	21 (4.6)	8 (2.2)	
Severe	0 (0.0)	1 (0.3)	
Mitral valve regurgitation			
None	48 (10.5)	37 (10.1)	0.872
Trace	149 (32.5)	123 (33.4)	
Mild	182 (39.7)	153 (41.6)	
Moderate	78 (17.0)	53 (14.4)	
Severe	2 (0.4)	2 (0.5)	
Mitral valve stenosis			
None	446 (97.2)	364 (98.9)	0.175
Mild	11 (2.4)	4 (1.1)	
Moderate	2 (0.4)	0 (0.0)	
Tricuspid valve regurgitation			
None	33 (7.2)	29 (7.9)	0.340
Trace	142 (30.9)	137 (37.2)	
Mild	178 (38.8)	130 (35.3)	
Moderate	92 (20.0)	61 (16.6)	
Severe	14 (3.1)	11 (3.0)	
Diastolic dysfunction according to the ASE/EACVI criteria (echocardiographic end point)^‡^	179 (52.8)	82 (32.0)	<0.001

Data with listwise deletion are expressed as mean (SD) or number (percentage).

* A single patient with missing sex data was excluded from the entire analysis.

^†^ Left atrial volume index and left ventricular ejection fraction was assessed with modified Simpson method.

^‡^ Diastolic dysfunction was diagnosed according to the American Society of Echocardiography and European Association of Cardiovascular Imaging (ASE/EACVI) guidelines for diastolic function assessment.^14^ A total of 595 patients had enough echocardiographic data for the assessment of diastolic dysfunction based on the criteria.

### Clinical End Point

**Figure 2:**
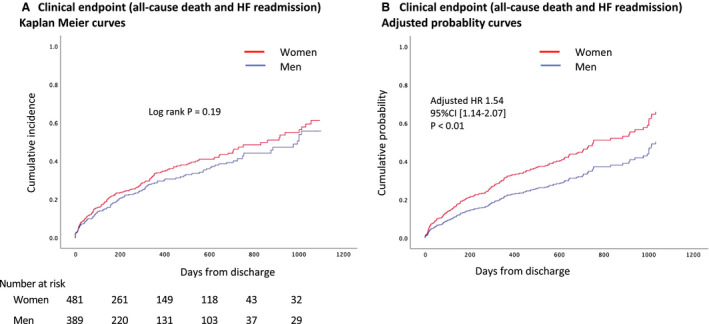
Clinical outcomes stratified by sex. **A**, The clinical end point of all‐cause death or heart failure readmission was assessed in a time‐to‐first‐event fashion with Kaplan–Meier analysis. In the crude comparison, no difference was found between women and men (log‐rank *P*=0.191). **B**, Adjusted probability curves in women and men created with the multivariable Cox proportional hazards model included the following covariates: female sex, C‐reactive protein, age, anemia (hemoglobin level <12 g/dL in women and <13 g/dL in men according to the World Health Organization definition^15^), hypertension, diabetes mellitus, dyslipidemia, coronary artery disease, chronic kidney disease, atrial fibrillation, obesity (body mass index ≥25), and cholinesterase level.^6^, ^13^ The cumulative probability curves show the model‐predicted event rates for the “average” patient in women and men. HF indicates heart failure; and HR, hazard ratio.

**Figure 3:**
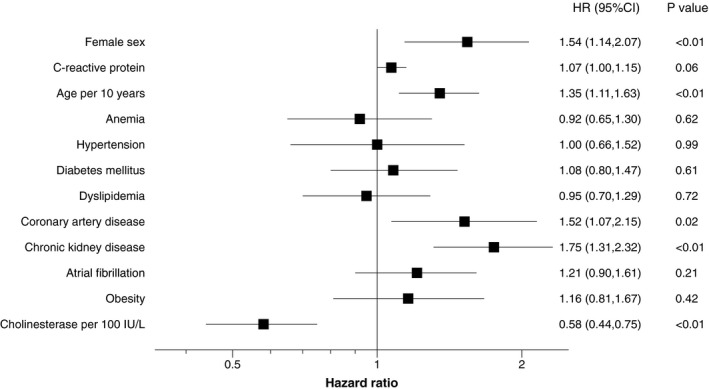
Prognostic factors for the clinical end point in the overall cohort. A multivariable Cox proportional hazards model was constructed in order to assess the impact of multiple comorbidities on the postdischarge clinical end point in the overall cohort (N=870). The results are shown as a forest plot. Female sex, age, coronary artery disease, chronic kidney disease, and cholinesterase were significantly associated with the clinical end point. HR indicates hazard ratio.

The clinical end point of all‐cause death or heart failure readmission occurred in 265 patients (30.5%) during the follow‐up period. The incidence of the clinical end point did not differ between women and men (women 36.1/100 person‐years versus men 30.5/100 person‐years, *P*=0.336) (Table 4). Kaplan–Meier curves and adjusted probability curves stratified by sex are presented in Figure 2. In the overall cohort, female sex, age, coronary artery disease, chronic kidney disease, and cholinesterase were independently associated with the clinical end point (Figures 2B and 3). Female sex was independently associated with increased risk of the clinical end point, which was mainly driven by the association with heart failure readmission (Table S1). Chronic kidney disease and cholinesterase were significantly associated with the clinical end point both in women and men, whereas coronary artery disease was an independent predictor only in women, although there were no significant interactions between the effect of the individual factors and sex (Figure S2). As a sensitivity analysis, we additionally constructed a Cox proportional hazards model including postdischarge medications (Figure S3). The result was consistent with the main analysis.

**Table 4:** Incidence of the Clinical End Points

Event	Women	Men	*P* Value
All‐cause death and heart failure readmission	36.1/100 person‐years	30.5/100 person‐years	0.336
All‐cause death	12.8/100 person‐years	12.8/100 person‐years	0.929
Cardiac death	6.1/100 person‐years	5.1/100 person‐years	0.544
Noncardiac death	6.7/100 person‐years	7.4/100 person‐years	0.601
Death from unknown cause	0/100 person‐years	0.3/100 person‐years	0.368
Heart failure readmission	24.1/100 person‐years	20.2/100 person‐years	0.426

## Discussion

The findings of this study can be summarized as follows: In the PURSUIT‐HFpEF prospective multicenter East‐Asian HFpEF registry, (1) women accounted for 55.2% of the overall cohort; (2) women had echocardiographic diastolic dysfunction more frequently than men; (3) female sex was independently associated with the presence of echocardiographic diastolic dysfunction; (4) crude incidence of the clinical end point of all‐cause death or heart failure readmission did not differ between women and men; (5) however, after multivariable adjustment, female sex was independently associated with increased risk of the clinical end point; and (6) there were no significant interactions between sex and the effects of comorbidities on echocardiographic and clinical end points.

### Diastolic Dysfunction in Women

Female sex was independently associated with diastolic dysfunction. This primary finding is supported by several previous studies.^20^, ^21^ A cross‐sectional study was conducted to examine sex differences in cardiometabolic profiles and exercise hemodynamic profiles among individuals with HFpEF.^20^ This cross‐sectional study included 295 participants who met hemodynamic criteria for HFpEF based on invasive cardiopulmonary exercise testing results. They examined sex differences in hemodynamic parameters during exercise with right heart catheterization. Exercise capacity was similar in men and women, but women had worse biventricular systolic reserve and diastolic reserve even after multivariable adjustment. The impaired diastolic reserve in women is not the same but correlated with diastolic dysfunction on echocardiography. Another study evaluated a total of 161 subjects using invasive hemodynamic and echocardiographic approaches.^21^ Compared with men, women had a higher pulmonary capillary wedge pressure indexed to peak exercise workload and lower systemic and pulmonary arterial compliance at exercise. Women had higher mitral inflow velocity to diastolic mitral annular velocity at early filling ratios at rest and peak exercise, along with a higher ejection fraction and smaller ventricular dimensions.

There was the entity of HFpEF without the echocardiographic diastolic dysfunction in the present study. This entity was more common in men than in women (68% in men versus 47% in women, *P*<0.001). Majority of this cohort may show impaired hemodynamics if they perform functional testing (eg, exercise stress echocardiography, invasive hemodynamic tests at rest and with exercise),^18^ because all participants were diagnosed with acute decompensated heart failure at the time of hospital admission. Given the previous evidence,^20^, ^21^ the potential population with impaired diastolic reserve during exercise but without the evidence of the echocardiographic diastolic dysfunction is presumably larger in women than in men. This would further contribute to the female preponderance in HFpEF. On the other hand, male patients had a higher prevalence of chronic kidney disease (46.0% versus 34.2%, *P*<0.001) and peripheral artery disease (8.0% versus 3.9%, *P*=0.011) than female patients did. These extracardiac deficits may more prominently affect systemic vascular resistance or abnormalities in peripheral oxygen extraction in men than in women.^22^, ^23^, ^24^ As for the cardiac function, deficits in contractile reserve rather than left ventricular diastolic dysfunction might play a more important role in men than in women.^23^ These points warrant further investigations.

The aforementioned sex‐specific cardiac features suggest that a kind of sex‐specific pathway exists. A variety of pathways has been thought to be associated with myocyte stiffness, including sex difference in calcium handling,^25^ myocardium substrate metabolism,^26^ an activated renin‐angiotensin‐aldosterone system in response to low estrogen,^27^ a drop in nitric oxide with menopause,^27^ protein kinase A,^28^ and extracellular signal‐regulated kinase 2 activated by progesterone.^29^ Sex‐specificity in patients with HFpEF is also likely supported by the heterogeneity with a possible benefit of sacubitril–valsartan seen in women in the PARAGON‐HF (Prospective Comparison of Angiotensin Receptor–Neprilysin Inhibitor with Angiotensin Receptor Blockers Global Outcomes in HF with Preserved Ejection Fraction) trial.^30^ Although our present study cannot provide a specific answer for the mechanism of HFpEF, our findings clearly suggest that future investigations of this condition should be sex specific.

In order to gain insight into the causes of diastolic dysfunction, we evaluated the association of various comorbidities with echocardiographic diastolic dysfunction. Systemic inflammation has been thought to be related to the development of diastolic dysfunction.^6^ However, C‐reactive protein was not independently associated with echocardiographic diastolic dysfunction in our population. Numerous studies have correlated inflammatory markers with diastolic dysfunction and HFpEF in humans.^31^ Nevertheless, our present results show no such impact. Anemia and obesity were independently associated with development of diastolic dysfunction. Anemia may partially be related to iron deficiency. It affects the immune response, cardiomyocyte metabolism, and oxidative stress.^32^ Another possibility is that anemia may be just a surrogate marker of multimorbidity. Whether this association is the result of specific shared upstream causes of both anemia and cardiomyocyte dysfunction (eg, inflammation) or causal relationships between HF and anemia (eg, decreased iron absorption) is unclear. In obesity, adipose tissue may exacerbate metabolic inefficiency and contribute to systemic inflammation.^33^ Although C‐reactive protein did not remain as an independent factor, this result does not reject the hypothesis that inflammation is a fundamental mechanism for the development of diastolic dysfunction. Unfortunately, the present study cannot provide enough data to answer this hypothesis. These topics need to be further investigated in basic science.

### Prognosis of HFpEF in Women and Men

Crude rates of the clinical end point of all‐cause death or heart failure readmission did not differ between women and men (Figure 2A). However, after adjustment of various confounders, female sex was independently associated with adverse clinical events in HFpEF (Figures 2B and 3). This may be a result of fewer baseline comorbidities in women than in men. Previous studies also reported that comorbidity burden in women is lower than that in men.^20^, ^34^ The primary finding is, however, inconsistent with the previous data from a large‐scale study (N=42 987) by Stolfo et al.^35^ In the Swedish Heart Failure Registry population, multivariate Cox and logistic regression models were fitted to investigate differences in prognosis, prognostic predictors, and treatments across men and women. Of 42 987 patients, 9957 patients had HFpEF. Crude mortality/HF hospitalization rates were significantly higher in women than in men (hazard ratio [HR], 1.14; 95% CI, 1.07–1.21). After adjustments, however, the risk was significantly lower in women (HR, 0.93; 95% CI, 0.88–0.99). Differences not only in the basic comorbidities but also in the postdischarge medications such as angiotensin II receptor blockers and calcium channel blockers between the sexes may have affected the clinical outcomes. In the study from the Swedish Heart Failure Registry, these medications were adjusted, whereas in our main analysis, we did not adjust the differences in these medications. However, our sensitivity analysis adjusting postdischarge medications provided consistent results (Figure S3). Racial difference would be one of the possible reasons for the opposite results between ours and the previous data. The difference in age (82±9 in the current cohort versus 79±10 in the Swedish Heart Failure Registry) and body mass index (22 versus 27) may also partially explain the opposite findings. This point remains to be further investigated in future studies.

### Clinical Implications

Sex differences in HFpEF suggest the need for further research to better understand underlying pathophysiology, including contributions of sex hormones and sex hormone deficiency, and thereby identify novel preventive and disease‐modifying treatments for HFpEF.

Anemia and obesity, besides female sex, were independently associated with diastolic dysfunction. Anemia or iron deficiency and weight control might be targets for preventing diastolic dysfunction. Besides female sex, coronary artery disease and chronic kidney disease were independently associated with worse clinical outcomes. Treatments for coronary artery disease and chronic kidney disease may be priorities in the treatments of HFpEF. Our results did not show significant interactions between sex and effects of any comorbidities (Figures S1 and S2). Therefore, aggressive therapeutic intervention for these comorbidities regardless of sex would be a reasonable option for the time being.

### Limitations

Several limitations should be acknowledged. First, the present study is a multicenter prospective East‐Asian HFpEF registry, which would limit the generalizability of the current findings to other races. The cutoff value for obesity (body mass index of 25 in the current analysis) would be different for other countries. Second, small sample size, especially of the subgroup analysis stratified by sex, might have resulted in type II error. Results should be interpreted with caution. Third, systemic inflammation was represented by C‐reactive protein in the current study. However, other inflammatory markers (interleukin‐6, tumor necrosis factor‐α, etc) should be investigated in future studies. Fourth, diastolic dysfunction was assessed only in patients with enough echocardiographic data (68% of the entire cohort). This might have resulted in selection bias. Lastly, the study demonstrated that female sex was independently associated with the presence of diastolic dysfunction. However, it is unclear whether the association of female sex with HFpEF is the result of innate biological differences (eg, sex hormones), the result of sex differences (environmental interactions that differ between sexes), or some other residual confounding (eg, women live longer than men). Future basic research would be mandatory to elucidate the specific mechanism of development of diastolic dysfunction in women.

## Conclusions

In the PURSUIT‐HFpEF prospective multicenter East‐Asian HFpEF registry, women accounted for 55.2% of the overall cohort. Women had echocardiographic diastolic dysfunction more frequently than men. Female sex was independently associated with the presence of diastolic dysfunction and worse clinical outcomes in a cohort of elderly patients with HFpEF. Our results suggest that a sex‐specific approach would be key to investigating the pathophysiology in HFpEF.

## Sources of Funding

This work was funded by Roche Diagnostics K.K. and Fuji Film Toyama Chemical Co. Ltd.

## Disclosures

Y. Sotomi received personal fees from Daiichi‐Sankyo, Bayer, Boehringer Ingelheim, and Bristol‐Myers Squibb. S. Hikoso received grants from Roche Diagnostics, FUJIFILM Toyama Chemical, Actelion Pharmaceuticals; personal fees from Daiichi Sankyo, Astellas Pharma, Bayer, Pfizer Pharmaceuticals, Boehringer Ingelheim Japan, Kowa Company, and Ono Pharmaceutical. D. Nakatani received personal fees from Roche Diagnostics. H. Mizuno is an endowed chair lecturer supported by Asahi Intecc Co., Ltd, Terumo Corporation, Nipro Corporation and Shimadzu Corporation, and received personal fees from Medtronic Japan Co.,Ltd, Japan Tobacco Inc, Pfizer Japan Inc., Bayer Yakuhin, Ltd., Japan Lifeline Co.,Ltd, Abbott Japan LLC., Nippon Boehringer Ingelheim Co., Ltd, Toa Eiyo Ltd, Daiichi Sankyo Co., Ltd, and Kowa Co., Ltd. K. Okada received personal fees from Bayer. Y. Sakata received personal fees from Otsuka Pharmaceutical, Ono Pharmaceutical, Daiichi Sankyo, Mitsubishi Tanabe Pharma Corporation, AstraZeneca K.K. and Actelion Pharmaceuticals, and received grants form Roche Diagnostic, FUJIFILM Toyama Chemical, Bristol‐Myers Squibb, Co, Biosense Webster, Inc., Abbott Medical Japan, Otsuka Pharmaceutical, Daiichi Sankyo Company, Mitsubishi Tanabe Pharma Corporation, Astellas Pharma, Kowa Company, Boehringer Ingelheim Japan, and Biotronik. The remaining authors have no disclosures to report.

## Supporting information


Appendix S1
Table S1
Figures S1–S3
