# Supplementary material for: Sex Differences in Heart Failure With Preserved Ejection Fraction
Source: J Am Heart Assoc. 2021 Feb 23;10(5):e018574. doi: 10.1161/JAHA.120.018574 (PMC8174270; doi:10.1161/JAHA.120.018574)

SUPPLEMENTAL MATERIAL

Appendix

The OCVC-Heart Failure Investigators

Chair: Yasushi Sakata, Department of Cardiovascular Medicine, Osaka University Graduate School of Medicine, 2-2 Yamada-oka, Suita 565-0871, Japan

Secretariat: Shungo Hikoso (Chief), Daisaku Nakatani, Hiroya Mizuno, Shinichiro Suna, Katsuki Okada, Tomoharu Dohi, Yohei Sotomi, Takayuki Kojima, Akihiro Sunaga, Hirota Kida, Bolrathanak Oeun, and Taiki Sato; Department of Cardiovascular Medicine, Osaka University Graduate School of Medicine, Suita, Japan.

Investigators:

Shunsuke Tamaki, Tetsuya Watanabe, and Takahisa Yamada, Osaka General Medical Center, Osaka, Japan; Takaharu Hayashi and Yoshiharu Higuchi, Osaka Police Hospital, Osaka, Japan; Masaharu Masuda, Mitsutoshi Asai, and Toshiaki Mano, Kansai Rosai Hospital, Amagasaki, Japan; Hisakazu Fuji, Kobe Ekisaikai Hospital, Kobe, Japan; Daisaku Masuda, Yoshihiro Takeda, Yoshiyuki Nagai, and Shizuya Yamashita, Rinku General Medical Center, Izumisano, Japan; Masami Sairyo, Yusuke Nakagawa and Shuichi Nozaki, Kawanishi City Hospital, Kawanishi, Japan; Haruhiko Abe, Yasunori Ueda, Masaaki Uematsu, and Yukihiro Koretsune, National Hospital Organization Osaka National Hospital, Osaka, Japan; Kunihiko Nagai, Ikeda Municipal Hospital, Ikeda, Japan; Masamichi Yano, Masami Nishino, and Jun Tanouchi, Osaka Rosai Hospital, Sakai, Japan; Yoh Arita and Shinji Hasegawa, Japan Community Health Care Organization Osaka Hospital, Osaka, Japan; Takamaru Ishizu, Minoru Ichikawa and Yuzuru Takano, Higashiosaka City Medical Center, Higashiosaka, Japan; Eisai Rin, Kawachi General Hospital, Higashiosaka, Japan; Yukinori Shinoda and Shiro Hoshida, Yao Municipal Hospital, Yao, Japan; Masahiro Izumi, Kinki Central Hospital, Itami, Japan; Hiroyoshi Yamamoto and Hiroyasu Kato, Japan Community Health Care Organization, Osaka Minato Central Hospital, Osaka, Japan; Kazuhiro Nakatani and Yuji Yasuga, Sumitomo Hospital, Osaka, Japan; Mayu Nishio and Keiji Hirooka, Saiseikai Senri Hospital, Suita, Japan; Takahiro Yoshimura and Yoshinori Yasuoka, National Hospital Organization Osaka Minami Medical Center, Kawachinagano, Japan; Akihiro Tani, Kano General Hospital, Osaka, Japan; Yasushi Okumoto and Hideharu Akagi, Kinan Hospital, Tanabe, Japan; Yasunaka Makino, Hyogo Prefectural Nishinomiya Hospital, Nishinomiya, Japan; Toshinari Onishi and Katsuomi Iwakura, Sakurabashi Watanabe Hospital, Osaka, Japan; Nagahiro Nishikawa and Yoshiyuki Kijima, Japan Community Health Care Organization, Hoshigaoka Medical Center, Hirakata, Japan; Takashi Kitao and Hideyuki Kanai, Minoh City Hospital, Minoh, Japan; Wataru

Shioyama and Masashi Fujita, Osaka International Cancer Institute, Osaka, Japan; Koichiro Harada, Suita Municipal Hospital, Suita, Japan; Masahiro Kumada and Osamu Nakagawa, Toyonaka Municipal Hospital, Toyonaka, Japan; Ryo Araki and Takayuki Yamada, Otemae Hospital, Osaka, Japan; Akito Nakagawa and Yoshio Yasumura, Amagasaki Chuo Hospital, Amagasaki, Japan; and Taiki Sato, Akihiro Sunaga, Bolrathanak Oeun, Hirota Kida, Takayuki Kojima, Yohei Sotomi, Tomoharu Dohi, Kei Nakamoto, Katsuki Okada, Fusako Sera, Shinichiro Suna, Hidetaka Kioka, Tomohito Ohtani, Toshihiro Takeda, Daisaku Nakatani, Hiroya Mizuno, Shungo Hikoso, Yasushi Matsumura and Yasushi Sakata, Osaka University Graduate School of Medicine, Suita, Japan.

Table S1. Association of the comorbidities and clinical endpoints

	**A composite of all-cause death and HF readmission***		**All-cause death***		**HF readmission†**	
	Hazard ratio [95%CI]	P value	Hazard ratio [95%CI]	P value	Subdistribution hazard ratio [95%CI]	P value
Female sex	1.538 [1.146, 2.064]	0.004	1.141 [0.733, 1.778]	0.560	1.553 [1.091, 2.211]	0.015
C-reactive protein	1.071 [0.976, 1.175]	0.150	1.150 [1.047, 1.263]	0.004	0.829 [0.716, 0.960]	0.012
Age	1.030 [1.010, 1.050]	0.003	1.083 [1.042, 1.125]	<0.001	1.008 [0.987, 1.031]	0.448
Anemia	0.916 [0.649, 1.292]	0.620	0.856 [0.481, 1.525]	0.600	1.045 [0.693, 1.576]	0.833
Hypertension	1.003 [0.690, 1.459]	0.990	0.776 [0.429, 1.402]	0.400	1.195 [0.715, 1.995]	0.497
Diabetes mellitus	1.083 [0.793, 1.480]	0.620	1.055 [0.641, 1.737]	0.830	1.004 [0.701, 1.437]	0.983
Dyslipidemia	0.946 [0.697, 1.284]	0.720	0.943 [0.571, 1.556]	0.820	1.123 [0.781, 1.613]	0.532
Coronary artery disease	1.518 [1.063, 2.167]	0.022	1.615 [0.973, 2.680]	0.064	1.392 [0.921, 2.104]	0.117
Chronic kidney disease	1.745 [1.318, 2.311]	<0.001	1.436 [0.939, 2.198]	0.095	2.062 [1.463, 2.907]	<0.001
Atrial fibrillation	1.206 [0.901, 1.614]	0.210	0.827 [0.527, 1.297]	0.410	1.418 [1.009, 1.994]	0.045
Obesity	1.161 [0.787, 1.712]	0.450	1.152 [0.646, 2.056]	0.630	1.008 [0.652, 1.556]	0.973
Cholinesterase	0.995 [0.992, 0.997]	<0.001	0.991 [0.986, 0.996]	0.001	0.996 [0.993, 0.999]	0.007

*Cox proportional hazard model was utilized to assess the impacts of the covariates on a composite of all-cause death and HF readmission, and all-cause death. †The Fine and Gray model was used for assessing subdistribution hazards for HF readmission considering all-cause death as a competing risk. Abbreviations: HF, heart failure, CI, confidence interval.

Figure S1. Comorbidities related to diastolic dysfunction in women and men

Multivariable binary logistic regression analysis was performed in order to assess the impact of multiple comorbidities on the echocardiographic endpoint (diastolic dysfunction) in women (**Red**) and men (**Blue**) separately. Results are illustrated as forest plot. In women, anemia was a unique and significant associated factor, whereas in men, there was no significantly associated factors with diastolic dysfunction. *P value for interaction between women and men.

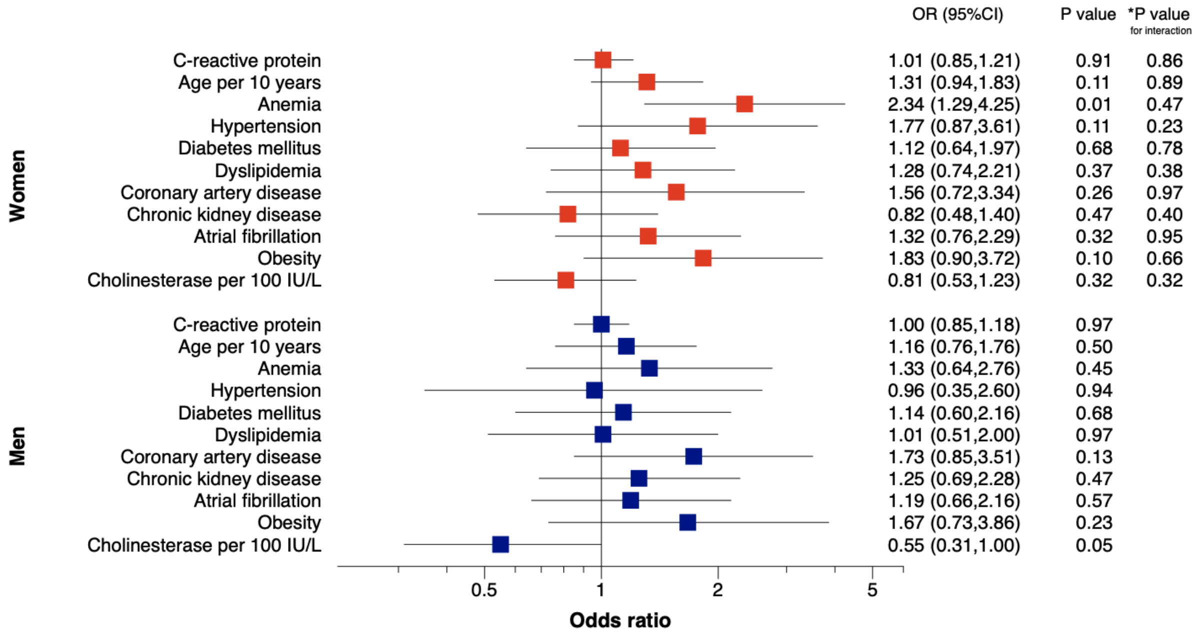

Figure S2. Prognostic factors for the clinical endpoint in women and men

Multivariable Cox proportional hazard model was constructed in order to assess the impact of multiple comorbidities on the post-discharge clinical endpoint in women (**Red**) and men (**Blue**) separately. Results are illustrated as forest plot. Chronic kidney disease and cholinesterase were significantly associated with the clinical endpoint both in women and men. Coronary artery disease was a significant predictor only in women, albeit no significant interaction. *P value for interaction between women and men.

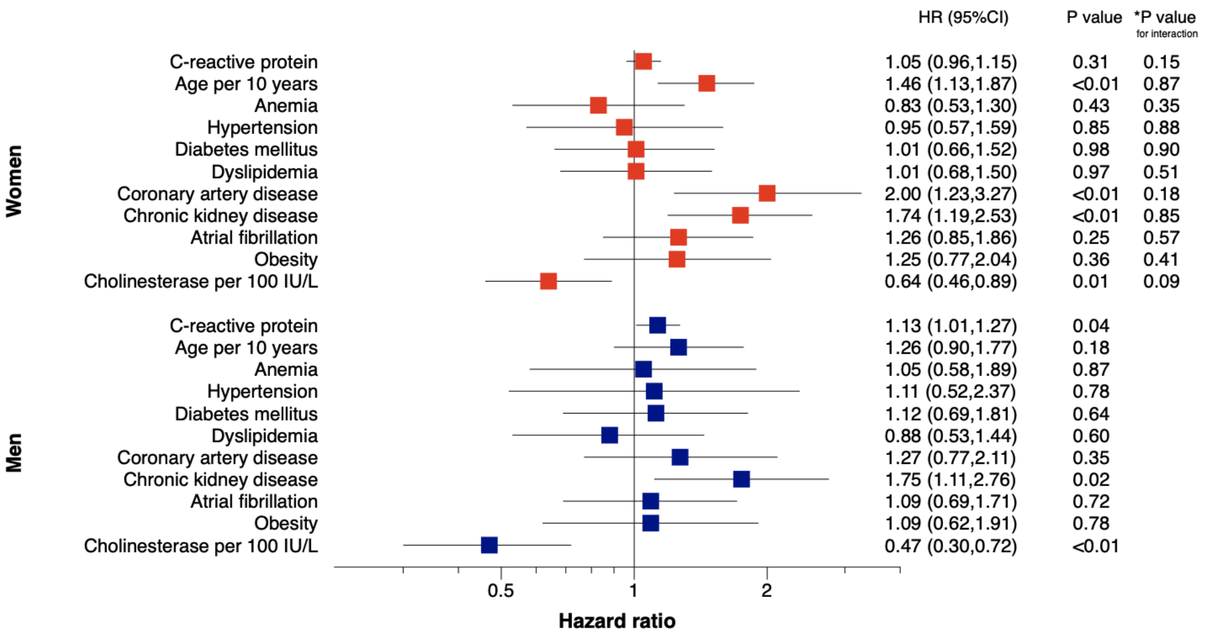

Figure S3. Prognostic factors for the clinical endpoint in the overall cohort

Multivariable Cox proportional hazard model was constructed in order to assess the impact of multiple comorbidities on the post-discharge clinical endpoint in overall cohort with adjustment for post-discharge medications. Results are illustrated as forest plot. Medications which prescription rates were different between women and men were included as covariates (**Table 2**). The result was consistent with the main analysis.

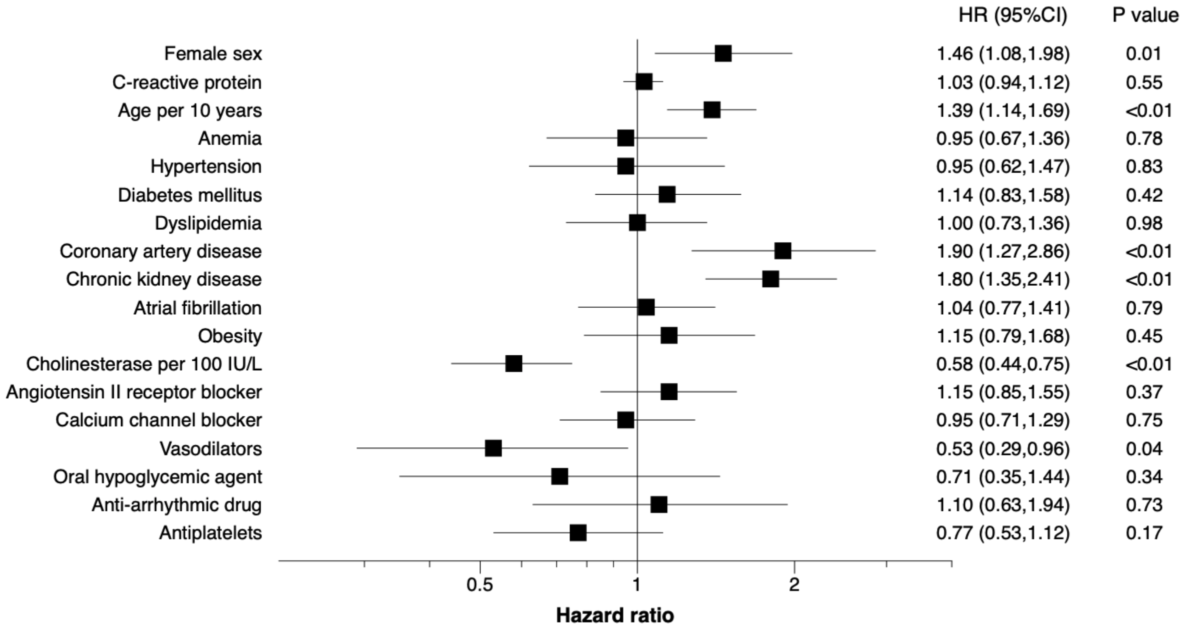

Supplement: Supplementary file 1 — Appendix S1 Table S1 Figures S1–S3 [file JAH3-10-e018574-s001.pdf]
